# Case report: When measured free T_4_ and free T_3_ may be misleading. Interference with free thyroid hormones measurements on Roche® and Siemens® platforms

**DOI:** 10.1186/1756-6614-5-11

**Published:** 2012-10-29

**Authors:** Krzysztof C Lewandowski, Katarzyna Dąbrowska, Andrzej Lewiński

**Affiliations:** 1 Department of Endocrinology and Metabolic Diseases, Polish Mother’s Memorial Hospital - Research Institute, Rzgowska St. No. 281/289, 93-338 Lodz, Poland; 2 Medical University, Lodz, Poland

**Keywords:** Free T_4_, Free T_3_, Assay interference

## Abstract

A 59-year old female patient presented with apathy and 6 kg weight gain. Investigations revealed severe primary hypothyroidism (TSH>100 μIU/ml). L-thyroxine (L-T_4_) was started and titrated up to 75 μg, once daily, with clinical improvement. Other investigations revealed very high titres of anti-thyroid peroxidase (anti-TPO) and anti-thyroglobulin (anti-Tg) antibodies. After three months, there was a fall in TSH to 12.74 μIU/ml, however, with unexpectedly high free T_4_ (FT_4_) - 6.8 ng/ml and free T_3_ (FT_3_) - 6.7 pg/ml concentrations [reference range (rr): 0.8-1.9 ng/ml and 1.5-4.1 pg/ml (Siemens®), respectively]. At this stage L-T_4_ was stopped, and this was followed by a rapid increase in TSH (to 77.76 μIU/ml) and some decrease in FT_4_ and FT_3_, however FT_4_ concentration remained elevated (2.1 ng/ml). Following this, L-T_4_ was restarted. On admission to our Department, she was clinically euthyroid on L-T_4_, 88 μg, once daily. Investigations on Roche® platform confirmed mildly elevated TSH - 5.14 (rr: 0.27-4.2 μIU/ml) with high FT_4_ [4.59 (rr: 0.93-1.7 ng/ml)] and FT_3_ [4.98 (rr: 2.6-4.4 pg/ml)] concentrations. Other tests revealed hypoechogenic ultrasound pattern typical for Hashimoto thyroiditis. There was no discrepancy in calculated TSH value following TSH dilution (101% recovery). Concentrations of FT_4_ and FT_3_ were assessed on the day of discontinuation of L-T_4_ and after four days by the means of Abbott® Architect I 1000SR platform. These revealed FT_4_ and FT_3_ concentrations within the reference range [e.g., FT_4_ - 1.08 ng/ml (rr: 0.7-1.48)] *vs* 4.59 ng/ml (rr: 0.93-1.7, Roche®), FT_3_ - 3.70 pg/ml (rr: 1.71-3.71) *vs* 4.98 (rr: 2.6-4.4, Roche®)], confirming assay interference. Concentrations of ferritin and SHBG were normal.

**Conclusions:**

Clinicians must be aware of possible assay interference, including the measurements of FT_4_ and FT_3_ in the differential diagnosis of abnormal results of thyroid function tests that do not fit the patient clinical presentation.

## Case presentation

Written informed consent was obtained from the patient for publication of this case report and any accompanying images. The publication in question was approved by the ethical committee of the Polish Mother’s Memorial Hospital – Research Institute. A 59-year old female patient presented with a history of apathy and weight gain (about 5–6 kg). Initial investigations revealed severe primary hypothyroidism (TSH>100 μIU/ml), moderate hyperlipidaemia (total cholesterol 239 mg/dl, triglicerides 185 mg/dl) and normal fasting glucose (83 mg/dl). She was started on L-thyroxine (L-T_4_), 25 μg, once daily, the dose of which was gradually titrated up to 75 μg, once daily, with clinical improvement. Other investigations revealed very high titre of anti-thyroid peroxidase antibodies (>4000 IU/ml (reference up to 115 IU/ml)). After about 3 months of treatment test revealed expected fall in TSH concentrations (to 12.74 μIU/ml), however, with unexpectedly high free thyroxine (FT_4_) and free triiodotyronine (FT_3_) concentrations (Siemens® platform - see Table 1). At this stage L-T_4_ was stopped, this was followed by a rapid increase in TSH (to 77.76 μIU/ml) and some decrease in FT_4_ and FT_3_, however, FT_4_ concentration still remained elevated. Following this, L-T_4_ was restarted. There was a gradual decrease in TSH, with FT_4_ and FT_3_ concentrations above the reference range. The patient was referred for the second opinion.

**Table 1:** **Initial results in the 59**-**year old female patient before and during treatment with L**-**T**_**4**_

**Date**	**TSH**	**FT**_**4**_	**FT**_**3**_	**L**-**T**_**4 **_**dose**	**Reference Range**
Initial results	>100	-	-	none	TSH 0.27-4.0 μIU/ml (Siemens®)
					FT_4_ 0.8-1.9 ng/ml (Siemens®)
					FT_3_ 1.5-4.1 pg/ml (Siemens®)
13-14 weeks	12.74	6.8	6.7	75 μg	
16 weeks	77.76	2.1	4.0	None for two weeks	
Seven months	49.23	3.1	4.5	50 μg	
Twelve months	8.41	4.8	5.6	88 μg	

On admission to our Department, she was clinically euthyroid. Her medication included L-T_4_, 88 μg, once daily, Rosuvastatin, 10 mg, once daily, Amlodipine, 5 mg x 1, once daily, Metoprolol slow release, 25 mg, once daily. There was no overt history of angina. She did not smoke and did not consume alcoholic beverages in excess.

Investigations performed in our Department (Roche® platform) confirmed mildly elevated TSH with high FT_4_ and FT_3_ concentrations (Table 2). The patient vehemently denied any compliance problems. Other tests confirmed very high titres of anti-thyroid peroxidase (anti-TPO) anti-thyroglobulin (anti-Tg) antibodies [anti-Tg > 4000.00 IU/ml (rr: up to 115 IU/ml); anti-TPO antibodies > 600.00 IU/ml (rr: up to 34 IU/ml)] and hypoechogenic ultrasound pattern typical for Hashimoto thyroiditis. Cortisol concentration was 10.52 μg/dl, ACTH 14.7 pg/ml (rr: 0–46 pg/ml). There was no discrepancy in calculated TSH value following TSH dilution (Table 2). Following this, L-T_4_ was stopped with assessment of thyroid function (Table 3). Again there was a gradual increase of TSH with some decrease in FT_4_ and FT_3_, however, still above the reference ranges. We also assessed FT_4_ and FT_3_ on the day of discontinuation of L-T_4_ and after 4 days in a different laboratory (Abbott® Architect I 1000SR platform – Table 3). This revealed FT_4_ and FT_3_ concentrations within reference ranges. Furthermore, the measured FT_4_ concentration was about 4 times lower than on the Roche® platform (Table 3). Concentrations of ferritin and SHBG were normal (Table 3). Following administration of a single dose of 300 μg, once daily, there a was a decrease in TSH with very little change of FT_4_ and FT_3_ (Roche® platform). Measurements of heterophilic antibodies were unfortunately not available in our Department. A diagnosis of hypothyroidism due to Hashimoto thyroiditis was made. The patient was discharged on L-T_4_, 100 μg, once daily. The patient, as well as her GP were informed about problems with FT_4_ and FT_3_ measurements, and that further adjustments of L-T_4_ dose should be based on clinical picture, as well as TSH measurements alone.

**Table 2:** **TSH dilution test in the 59**-**year old female patient on 88** μ**g of L**-**T**_**4 **_**daily**

**Dilution**	**TSH**	**FT**_**4**_	**FT**_**3**_	**Reference Range**
none	4.37	6.61	5.57	TSH 0.27-4.2 μIU/ml (Roche®)
				FT_4_ 0.93-1.7 ng/ml (Roche®)
				FT_3_ 2.6-4.4 pg/ml (Roche®)
1:5	0.885	-	-	
Calculated TSH	5 x 0.885=4.42 (101.1% recovery)	-	-	

**Table 3:** **Results of thyroid function tests in the 59**-**year old female patient during L**-**T**_**4 **_**withdrawal**

**Date**	**TSH**	**FT**_**4**_	**FT**_**3**_	**Ferritin**	**SHBG**	**Reference Ranges**
14.06.2012#	5.14	4.59	4.98	79.9		TSH 0.27-4.2 μIU/ml (Roche®)
						FT_4_ 0.93-1.7 ng/ml (Roche®)
						FT_3_ 2.6-4.4 pg/ml (Roche®)
14.06.2012	not repeated: Roche® platform	1.08*	3.70*		79.5	*Abbott® Architect I 1000SR
						FT_4_: 0.7-1.48 ng/dl
						FT_3_: 1.71-3.71 pg/ml
15.06.2012	6.5	4.5	4.97			Ferritin: 5–148 ng/ml
16.06.2012	9.49	3.48	4.82			SHBG: 18.8-115.2 nmol/l
18.06.2012	11.01	3.41	4.74	81.00		
18.06.2012##	not repeated: Roche® platform	1.00*	3.38*		69.5	*Abbott® Architect I 1000SR
						FT_4_: 0.7-1.48 ng/dl
						FT_3_: 1.71-3.71 pg/ml
19.06.2012	9.53	3.57	4.74			

# L-T_4_ stopped on this day.

## 300 μg of L-T_4_ administered after blood test on 18.06.2012.

## Discussion

Measurements of thyrotropin (TSH) and of total and FT_4_ and FT_3_ are widely used for thyroid function evaluation. However, some serum samples might demonstrate a nonspecific binding with assay reagents that can interfere with the measurement of these hormones. Several authors have reported the presence of such interferences, resulting in abnormal concentrations of thyroid hormones inconsistent with the patient's clinical picture [1-3]. Interference in immunoassays is a widely recognized problem, which could potentially lead to unnecessary investigations and treatment. We describe a case, with interference in the FT_4_ and FT_3_ assay that led to falsely elevated serum FT_3_ and FT_4_ concentrations.

Clinical and biochemical investigations of our patient showed that biochemical thyroid status did not match her clinical presentation. Namely, the patient had high FT_4_ and FT_3_ levels with grossly elevated TSH, while simultaneously she did not have clinical features of hyperthyroidism. Further investigations revealed that she had very high titres for anti-TPO antibodies and anti-Tg antibodies, as well as thyroid ultrasound pattern typical for Hashimoto thyroiditis. Moreover, following treatment with L-T_4_, we observed an expected fall in TSH concentrations, accompanied by clinical improvement, but also accompanied by further increase of FT_4_ and FT_3_, far above the reference ranges. On the other hand, L-T_4_ withdrawal resulted in fast increase in TSH, with only gradual decrease in FT_4_ and FT_3_, where FT_4_ still remained above the reference range, despite very high TSH concentration (see Table 1). For these reasons, we considered assay interference. Therefore, we carried out dilution in TSH serum, which demonstrated linear correlation and almost 100% recovery. Manufacturer’s instructions precluded dilution tests with FT_4_ and FT_3_ assays. Such situation was different from recently described falsely elevated TSH level due to macro-TSH [4]. Furthermore, investigations in our Department confirmed an expected rise of TSH after L-T_4_ withdrawal.

In our patient, based on biochemical studies alone, the differential diagnosis also included thyroid hormone resistance syndrome or a TSH-oma. Both entities are characterized by clinical thyrotoxicosis, diffuse goiters, elevated circulating levels of FT_4_ and FT_3_, and non-suppressed serum TSH, though such very high initial TSH concentrations (i.e., above the upper limit of TSH assay), are not typical for these entities. However, given significant clinical improvement on L-T_4_ treatment, high antibody titres, as well as thyroid ultrasound picture typical for Hashimoto thyroiditis, we had decided not to perform either a TRH test, or pituitary MR scanning. We are aware of the fact that in case of thyroid hormone resistance or a TSH-oma one might expect marked and progressive worsening of thyrotoxicosis following gradual increase in the dose of L-T_4_. Moreover, we also investigated concentrations of SHBG and ferritin in serum. Thyroid hormone is one of several factors that modulate the level of sex hormone-binding globulin (SHBG) in serum. SHBG levels are usually elevated in thyrotoxicosis and have been reported to be normal in a few patients with generalized resistance to thyroid hormone (GRTH) [5,6]. Also, a significant body of evidence exists showing a positive correlation between the serum levels of T_3_, T_4_ and ferritin [7,8] in patients with abnormal thyroid status. Namely, all of these studies documented elevated serum ferritin levels in patients with hyperthyroidism which normalized when the T_3_ and T_4_ levels returned to normal. In our patient levels of SHBG and ferritin were normal.

For these reasons we concluded that assay interference was the only plausible explanation for the observed abnormalities. As direct test for heterophilic antibodies was not available in our institution, then we decided to repeat the blood tests using a different assay (i.e. two step assay – Abbott Architect®) in contrast to one step Siemens® and Roche® assays. This showed markedly lower concentration of FT_4_ and also lower concentration of FT_3_, consistent with the clinical picture. A possibility of assay interference is often under-recognised in clinical practice, while some patients’ sera contain autoantibodies to thyroid hormone that result in methodological artifacts in total or free hormone measurements [9,10]. In such case, tracer T_4_ or T_3_ binds to the endogenous heterophilic antibody and is subsequently falsely classified as bound to the sought substance by adsorption methods, or alternatively - falsely classified as free by double antibody methods, leading to falsely low or falsely high serum total T_4_ or total T_3_ values, respectively [10,11]. The T_4_ or T_3_ tracer analogs, used in some FT_4_ or FT_3_ tests, may also bind to these autoantibodies, leading to spuriously high serum FT_4_ and FT_3_ results. Interference in thyroid hormones assays may thus cause a mismatch between clinical and biochemical data and lead to diagnostic dilemmas and inappropriate treatment. Heterophilic antibodies are antibodies produced against poorly defined antigens. They are generally weak antibodies with multi-specific activities. Heterophilic antibodies are present in 30-40% of the population. However, they seldom cause interference with diagnostic immunoassays (0.05-0.5%), unless present in particularly high titres [12]. By definition, heterophilic antibodies are directed against specific animal immunoglobulins or against immunoglobulins of various animal species, depending on the recognized epitope and on the cross-reactivities between species immunoglobulins [10,13]. The recent development of two-site immunometric assays with specific antibodies, such as mouse monoclonal antibodies, has enabled higher specificities and sensitivities. Since the introduction of these assays, there have been several reports of abnormal concentrations of TSH resulting from heterophilic antibody interference [10]. To counteract this problem, all commercial assays now include blocking reagents, such as nonspecific and polymerized murine IgG. However, the presence of blocking reagents does not completely eliminate the problem of interference in some specimens and with some kits.

The FT_4_ and FT_3_ assays in our lab used a kit produced by Roche Diagnosis GmbH, Mannheim (sandwich Elecsys). Elecsys is an electrochemiluminescent immunoassay (ECLIA) involving ruthenium as the luminescent material. To exclude interference, the thyroid function tests were performed again, using the Architect system (Abbott). The Architect FT_3_ and FT_4_ assay is a two-step immunoassay determining the presence of free (unbound) T_3_ and T_4_ in human serum and plasma using chemiluminescent microparticle immunoassay (CMIA). Unfortunately, as we mentioned before, direct tests to detect particularly high titres of heterophilic antibodies were not available in our Department. However, given the patient's clinical condition, results of other investigations, as well as significant improvement in comfort after treatment with L-T_4_, we concluded that the cause of disharmony between clinical picture and biochemical results was related to FT_4_ and FT_3_ assay interference.

## Conclusion

Clinicians need to be aware of the potential for interference in immunoassays by heterophilic antibodies this since such inaccurate results of thyroid function tests may lead to inappropriate treatment decisions.

## Competing interests

The authors declare that they have no competing interests.

## Authors contributions

KCL was a senior physician responsible for patient’s treatment and he prepared the manuscript, KD was a medical resident responsible for patient’s care, AL was a senior author, he conceived the study and revised the text of manuscript. All authors have read and approved the final manuscript.
